# Characterization of liposomal carriers for the trans-scleral transport of Ranibizumab

**DOI:** 10.1038/s41598-017-16791-7

**Published:** 2017-12-01

**Authors:** Rini Rachel Joseph, Dulcia Wei Ni Tan, Moreno Raja Miguel Ramon, Jayaganesh V. Natarajan, Rupesh Agrawal, Tina T Wong, Subbu S Venkatraman

**Affiliations:** 1 School of Materials Science and Engineering, Nanyang Technological University, Singapore, Singapore; 2 Singapore National Eye Centre, Singapore, Singapore; 3 Ocular Drug Delivery Group, Singapore Eye Research Institute, Singapore, Singapore; 4 National Healthcare Group Eye Institute, Tan Tock Seng Hospital, Singapore, Singapore

## Abstract

Age-related macular degeneration (AMD) is a leading cause of blindness in the modern world. The standard treatment regimen for neovascular AMD is the monthly/bimonthly intravitreal injection of anti-VEGF agents such as ranibizumab or aflibercept. However, these repeated invasive injections can lead to sight-threatening complications. Sustained delivery by encapsulation of the drug in carriers is a way to reduce the frequency of these injections. Liposomes are biocompatible, non-toxic vesicular nanocarriers, which can be used to encapsulate therapeutic agents to provide sustained release. The protein encapsulation was performed by a modified dehydration-rehydration (DRV) method. The liposomes formed were characterized for size, zeta potential, encapsulation efficiency, stability, *in vitro* release, and *ex vivo* release profiles. In addition, the localization of the liposomes themselves was studied *ex vivo*. Entrapment-efficiency of ranibizumab into 100-nm liposomes varied from 14.7 to 57.0%. Negatively-charged liposomes prepared from DPPC-DPPG were found to have the slowest release with a low initial burst release compared to the rest of liposomal formulations. The *ex vivo* protein release was found to slower than the *in vitro* protein release for all samples. In conclusion, the DPPC-DPPG liposomes significantly improved the encapsulation and release profile of ranibizumab.

## Introduction

For several proliferative vitreoretinal diseases in the eye, intravitreal injection of anti-VEGF drugs is the current standard of treatment. This is an invasive procedure and may lead to sight-threatening complications such as endophthalmitis, cataract or even retinal detachment^1,2^.

The periocular route of ocular drug delivery has been proposed as a less invasive alternative to the posterior delivery with no disruption to the integrity of the eyeball^3^. The main barriers to trans-scleral delivery have been classified as static, dynamic and metabolic^4^. Transport characteristics of several drugs and macromolecules across the sclera have been studied over the past few decades to delineate the properties affecting the transport across this barrier. Notably, Ambati *et al*. have studied the transport of several small molecules across the sclera in an *ex vivo* setup to determine the size effect of these molecules on trans-scleral transport^5^. Several factors influence the transport of drugs to the posterior ocular segment, such as size, charge and lipophilicity^6–11^.

Chronic disorders affecting the posterior segment require extended treatment duration, and hence, it is desirable to develop sustained release systems. Various attempts have been made to improve therapeutic effect of drugs to the posterior segment, by the use of inserts, implants, micro- and nano-particulate systems, etc.^12,13^. Most of these formulations only focused on intravitreal sustained drug release^14–20^. However, very few reports were on subconjunctivally administered sustained release formulations. Sustained drug action in the subconjunctival space is challenging because of blood and lymphatic clearance mechanisms that wash out drugs^21^. Nevertheless, our group has demonstrated sustained duration of action for an anti-glaucoma drug via the sub-conjunctival route using nanoliposomes^22,23^, even though the drug target is in the anterior segment in this case.

Nanocarriers such as liposomes are particularly attractive owing to their biocompatibility, ability to deliver both hydrophobic and hydrophilic drugs as well as their non-toxic nature. Their size, charge, membrane rigidity and encapsulation efficiency are easily tunable. Although it is not trivial to achieve sustained release from nanoliposomes, our group has demonstrated that it is achievable with partition-controlled release^22^ for lipophilic drugs and another group has demonstrated it for a soluble drug such as gentamicin^24^.

In this study, we encapsulated ranibizumab into different lipid compositions, and characterized these liposomal compositions for their size, charge and encapsulation efficiency. *In vitro* and *ex vivo* release experiments were conducted as well. Finally, we also investigated the distribution and localization of the carriers themselves intrasclerally using an *ex vivo* setup.

## Results

### Ranibizumab-loaded Liposomes Characterization

Liposomes were extruded through polycarbonate membranes to achieve a size of approximately 100 nm. Table 1 shows the size and charge distribution of different liposome dispersions. The addition of cholesterol caused an increase in particle size in comparison to the rest of the liposome formulations. The samples were stored in 4 °C and the physical stability of the liposomes was investigated by measuring the changes of size after two weeks. No significant change in size was observed, except for DPPC-DPTAP sample, which increased 4 times its size, probably due to agglomeration of liposomes.

**Table 1:** Size and charge of ranibizumab-loaded liposomes.

Liposomal Formulation	Size (nm) (PDI)	Charge (mV)	Size after 2 weeks storage at 4 °C
DPPC	100.2 (0.07)	—	102.7 (0.10)
DPPC-Chol (4:1 mol/mol)	134.0 (0.19)	—	177.9 (0.42)
DPPC-DPTAP (4:1 mol/mol)	110.1 (0.20)	+39.1 (12.6)	442.1 (0.47)
DPPC-DPPG (4:1 mol/mol)	105.5 (0.06)	−38.9 (10.2)	107.9 (0.07)

**Figure 1:**
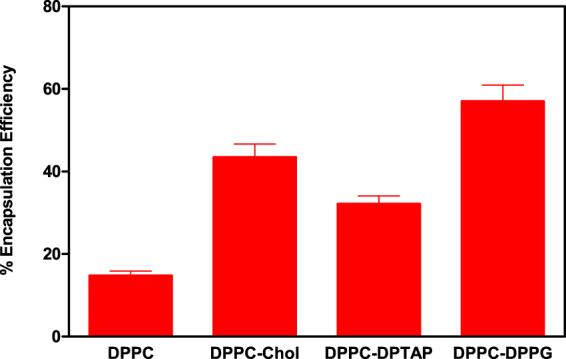
Comparison of the effect of liposome characteristics in encapsulation efficiency of liposomes.

The efficacy of entrapment of ranibizumab into liposomes were significantly different (Fig. 1). The lowest encapsulation efficiency was found for the DPPC liposomes (14.8 ± 1.1%), however when cholesterol was added, the efficacy of entrapped protein more than doubled. Interestingly, when positive (DPTAP) or negative (DPPG) phospholipids were incorporated to the lipid bilayer, loading capacity increased as well. The best ranibizumab entrapment efficiency achieved was the liposomal composition DPPC-DPPG (4:1), reaching around 60% (w/w). This high efficacy of ranibizumab entrapment could be due to the electrostatic interaction between the positive charged protein and the negative charged bilayer of liposomes. The buffer used to entrap ranibizumab was based on the commercial storage buffer consisting of 10 mM histidine-HCl, 10% α,α-trehalose dihydrate, 0.01% polysorbate 20, that has a pH of 5.5. Therefore, taking into account that ranibizumab’s isoelectric point is around 8.8^25^, at pH 5.5 the protein is positively charged.

### *Ex Vivo* Liposome Transport

**Figure 2:**
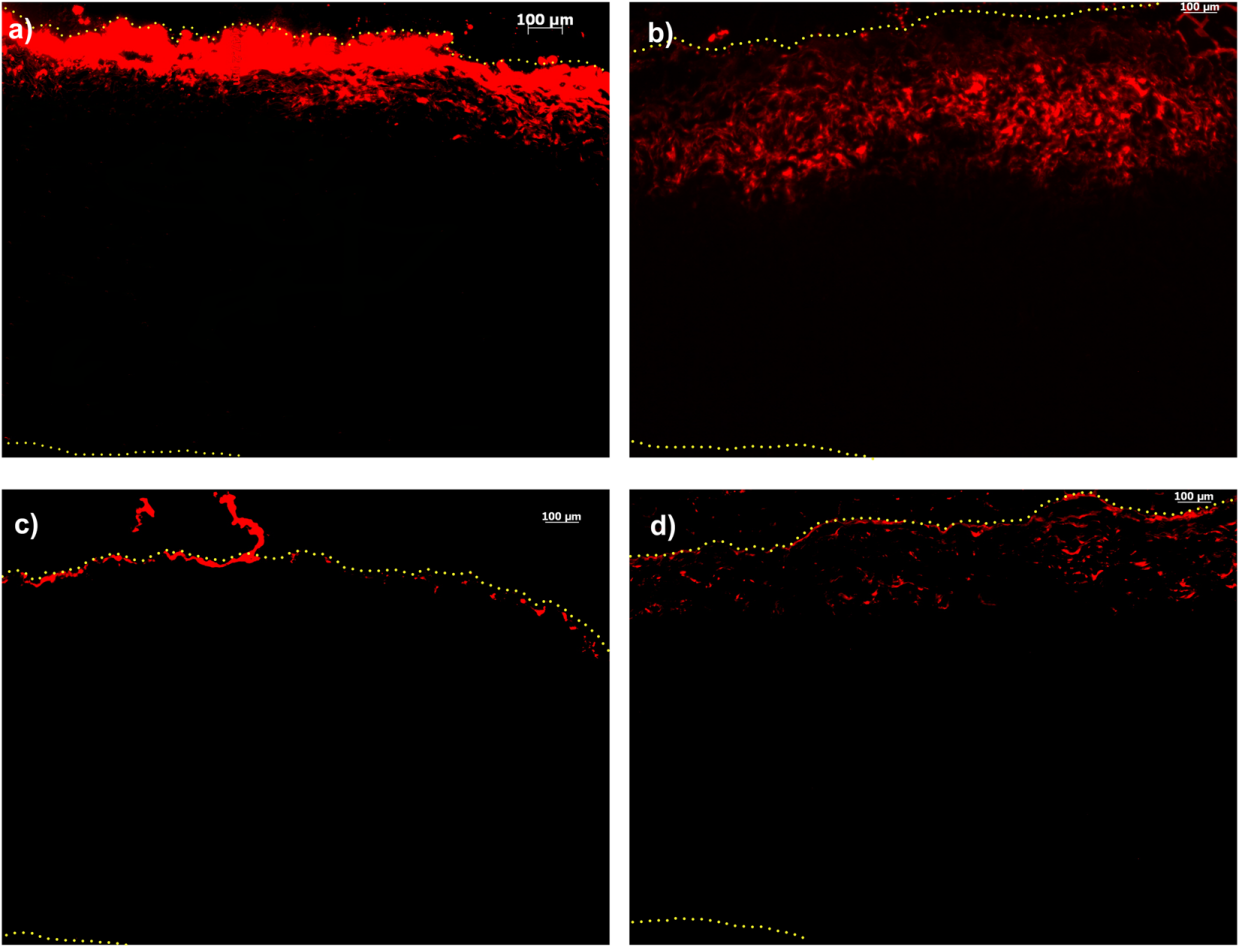
*Ex vivo* distribution of liposomes into the porcine sclera after 96 hours of incubation: (**a**) DPPC (**b**) DPPC-Cholesterol (4:1 molar ratio) (**c**) DPPC-DPTAP (4:1 molar ratio) (**d**) DPPC-DPPG (4:1 molar ratio). The edges of the tissue are marked using a dotted line, and the liposomes are fluorescently labeled with rhodamine PE (red).

**Figure 3:**
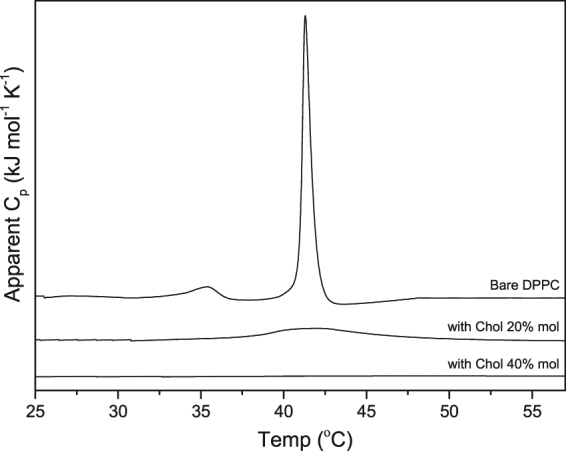
Thermogram of liposomes: Bare DPPC and DPPC with 20% mol and 40% mol cholesterol.

The *ex vivo* study was conducted for 96 hours with four formulations: DPPC, DPPC-Chol, DPPC-DPTAP and DPPC-DPPG. The results of *ex vivo* transport of liposomes across the porcine sclera are shown in Fig. 2. The DPPC liposomes localized preferentially close to the episcleral region, whereas the DPPC-Chol formulation showed higher degree of intrascleral diffusion compared to the DPPC formulation. Cholesterol is often incorporated to increase the rigidity of liposomal membrane. However, this only holds true for unsaturated lipids such as POPC, and not for DPPC, which is a saturated lipid. The addition of cholesterol to DPPC actually increases the chain flexibility, leading to a leakier bilayer structure^26^. We investigated the effect of cholesterol on DPPC using differential scanning calorimetry (DSC). In the absence of cholesterol, DPPC heating scan shows two sharp endothermic peaks at 34 °C and 41.2 °C with the first peak corresponding to the pre-transition (Lβ’/Pβ’) and the second peak to the main phase transition (Pβ’/Lα)^27^. At 20 mol% and 40 mol% cholesterol concentrations, the pre-transition peak disappears (Fig. 3). Furthermore, the main phase transition was significantly broadened in presence of 20 mol% cholesterol and was completely abolished at 40 mol% cholesterol; showing the vesicle rigidity is also determined by the amount of cholesterol added. The charge of liposomes had a clear effect on transport. Positively charged DPPC-DPTAP formulation was localized preferentially at the episcleral space and showed no appreciable transport into the sclera. On the other hand, negatively charged DPPC-DPPG showed to some extent episcleral penetration and transport.

### *In Vitro* Release

**Figure 4:**
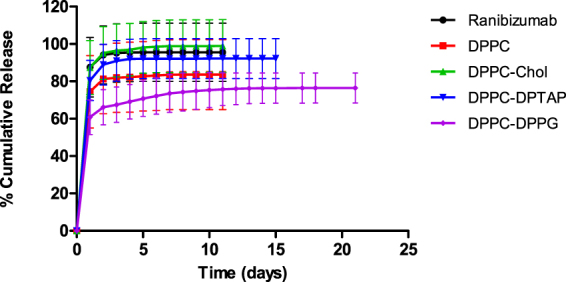
*In vitro* release profile of the different Ranibizumab loaded liposomal formulations.

An *in vitro* study was conducted, whereby the release profile of four different formulations was compared with the ranibizumab control (Fig. 4). The control formulation, DPPC and DPPC-Chol release were performed until Day 11, whereas releases of DPPC-DPTAP and DPPC-DPPG were continued till Day 15 and Day 21 respectively (until the amounts released were within the assay detection limits). Without a carrier for encapsulation, bare ranibizumab eluted out quickly from the dialysis bag. The incorporation of cholesterol to DPPC (DPPC-Chol) resulted in an overall increase to the release profile compared to that of DPPC alone. This could be due to drug localization on the surface of liposomes, as well as increased solute permeability due to decreased membrane rigidity by addition of cholesterol. DPPC-Chol showed a higher release of encapsulated contents with an initial release of 87.5 ± 14.4% compared to DPPC’s initial release of 74.4 ± 19.4%. Out of all the formulations, DPPC-DPPG exhibited the lowest burst of 60.8 ± 9.3%.

### *Ex Vivo* Transport of Ranibizumab-loaded Liposomes

**Figure 5:**
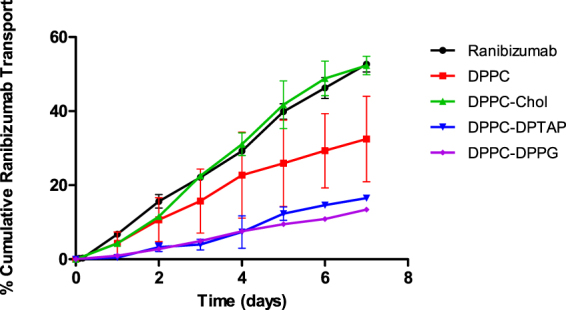
*Ex vivo* transport of Ranibizumab loaded liposomal formulations across seven days. The liposomes localized at the episcleral area showed some degree of intrascleral diffusion while the Ranibizumab from the loaded liposomes was able to diffuse trans-sclerally.

The *ex vivo* transport of bare ranibizumab was compared with ranibizumab encapsulated in DPPC, DPPC-Chol, DPPC-DPTAP and DPPC-DPPG liposomal formulations. Bare ranibizumab in the *ex vivo* setup was found to display a linear transport with 52.7 ± 2.1% of the drug transported and quantifiable at the end of the study. The comparison of transport between the formulations is depicted in Fig. 5. DPPC displayed a more controlled and sustained transport of ranibizumab than DPPC-Chol which showed a higher *ex vivo* transport as well. There was no significant difference observed between DPPC and bare ranibizumab. However, the charged DPPC-DPTAP and DPPC-DPPG formulations showed statistically significant difference in their *ex vivo* release profiles, compared to DPPC or bare ranibizumab formulations. Both the formulations exhibited a slower release compared to the other formulations. All formulations, except DPPC-Chol were found to have statistically significant (p < 0.05) difference in the *ex vivo* transport profile when compared with bare ranibizumab.

### *In vitro* vs *Ex Vivo* comparison

**Figure 6:**
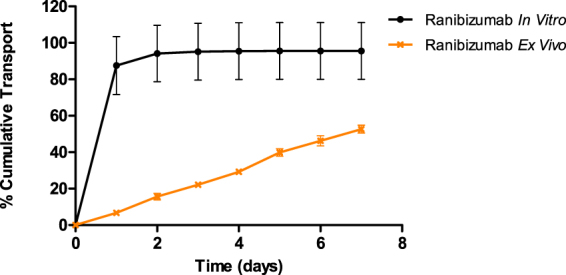
Comparison of bare ranibizumab *in vitro* and *ex vivo* setup. Each experiment was repeated in triplicates.

We saw a slower overall transport of ranibizumab across the sclera than is suggested by the *in vitro* release of ranibizumab. In contrast to the 100% transport of ranibizumab across the dialysis membrane (which we call *in vitro* “release”) in 2 days, the *ex vivo* study showed only about 50% of the drug was transported across the sclera (Fig. 6). This shows the potential of the sclera in acting as a depot and capable of providing sustained release from the subconjunctival/episcleral space.

**Figure 7:**
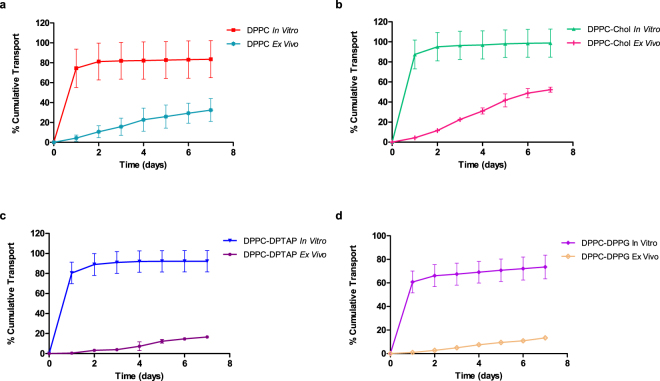
Comparison of *in vitro* release and *ex vivo* transport of the different liposomal formulations. Each experiment was repeated in triplicates.

The amount of drug evaluated in the *in vitro* as well as the *ex vivo* experiments were the same. The studies were not carried out for a longer time period due to the limitations of scleral tissue viability. In Fig. 7, the comparison of *in vitro* and *ex vivo* release from all four liposomal formulations are depicted.

All liposomal formulations showed slower transport across the sclera (*ex vivo*) with minimal burst compared to transport across the dialysis membrane (*in vitro* “release”). DPPC-DPPG formulation exhibited the slowest release/transport among the formulations. This could be attributed to the negatively charged DPPG interacting electrostatically with the ranibizumab, which exhibits a slightly global positive charge at pH 7.4. The electrostatic interaction of the drug with the sclera (as seen by the slower transport of ranibizumab by itself across the sclera compared to its diffusion across dialysis membrane) is an added advantage for trans-scleral transport. All formulations from *in vitro* release exhausted their contents by the second day, with most of the ranibizumab released as a burst on the first day. The *ex vivo* transport, however, showed a linear transport trend for seven days, which implies that the sclera acts as a classic membrane for diffusion of the ranibizumab.

## Discussion

**Figure 8:**
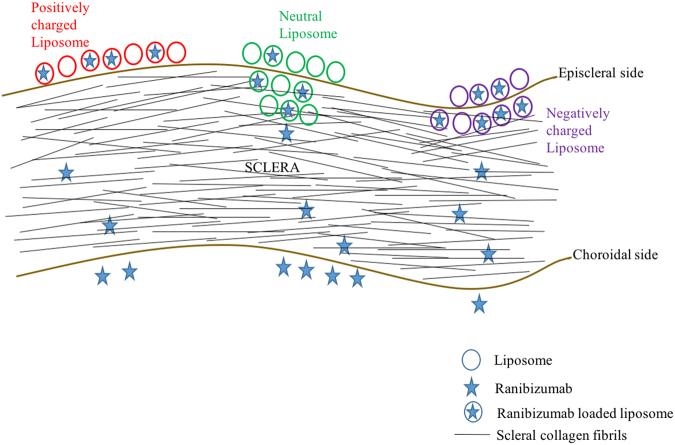
Schematic depicting the behavior of liposomes. Neutral liposomes localize at the surface mostly, and penetrate to a small extent, releasing ranibizumab which is then transported trans-sclerally. On the other hand, negatively charged liposomes penetrate intrasclerally and release the ranibizumab which then diffuses across the sclera. Positively charged liposomes, on the other hand remain localized episclerally and release the ranibizumab which diffuses across the sclera.

Intravitreal injections of liposomal formulations have shown promise in controlled delivery for some drugs^14,28–30^. Injection into the subconjunctival space is less invasive and offers the possibility of delivering multiple types of sustained release formulations to the eye such as liposomes. The goal of the present study was to entrap ranibizumab into different lipid formulations to control its release for sub-conjunctival administration. We observed a high ranibizumab entrapment efficiency into negatively charged liposomes made of DPPC-DPPG (∼60%). Therefore, the global positive charge of ranibizumab, in entrapment conditions at pH 5.5, enhanced the efficacy of protein entrapment. *In vitro* release studies showed burst release for ranibizumab in the first 24 hours for all liposomal formulations with no significant protein release in subsequent days. However, *ex vivo* transport experiments showed slower ranibizumab transport kinetics in comparison with *in vitro* results. This linear diffusional transport of the protein implies that the sclera is the rate-determining “membrane” for transport of the ranibizumab from the sub-conjunctival space to the back of the eye. For the negatively- charged liposome (DPPC-DPPG), we observed slower transport *ex vivo* than release *in vitro*. For the 7-day studies, the p value was found to be significantly different for the *ex vivo* transport and *in vitro* release (two-tailed t-test; p-value = 0.009; p < 0.05). The differences in transport for the different liposomes studied here are attributed to the depth of penetration in the sclera as well as the differences in release kinetics. If the carrier liposome accumulates at the surface of the sclera, and releases its cargo immediately (in 24 hours), then the released ranibizumab transports across the sclera via diffusion. However, when the carrier penetrates deeper with intact cargo, the ranibizumab releases slower overall from the carrier to the surrounding collagenous layer. The daily transport rate for ranibizumab in a negatively charged DPPC-DPPG *ex vivo* was about 2%, showing a more consistent daily release compared to positively charged DPPC-DPTAP. Figure 8 below shows a schematic depicting the scleral transport behavior of different charged liposomal carriers.

In a previous study, Kim *et al*.^31^ compared the diffusion of different drugs through the sclera from PLGA nanoparticles and liposomes. The authors concluded that the carriers do not diffuse through the sclera. However, our studies demonstrated the distribution of the liposomal nanocarriers inside the sclera, which can be further useful with drug loaded liposomes, with the carrier acting as a depot inside the sclera. The differences in the diffusional transport of fluorescent liposomes observed are due to size, charge and presence of cholesterol. In our study involving *ex vivo* transport of fluorescently tagged liposomes, we observe that DPPC liposomes are able to diffuse to a small extent into the sclera. The positive charged DPPC-DPTAP liposomes basically localized on the episcleral region due to electrostatic attraction with the negatively charged sclera. The negatively charged DPPC-DPPG liposomes were able to demonstrate some degree of scleral diffusion in the *ex vivo* condition, but less than zwitterionic liposomes, because of repulsion with the negatively charged sclera.

The permeability of liposomal membrane is one of the crucial factor that influences the release of encapsulated drug from liposomes and the influence of cholesterol on the thermotropic phase behavior of DPPC was found to affect the *ex vivo* transport and the *in vitro* release profiles. Cholesterol has the ability to disrupt the gel-state organization of DPPC and increase the probability of content leakage. The incorporation of cholesterol to saturated lipids like DPPC increases the permeability of liposomal membrane^32^. A study done by Takechi-Haraya *et al*. found cholesterol to be detrimental to saturated lipids, causing an increase in the permeability coefficient. The rigidity of the liposome membrane was decreased, allowing encapsulated drug to elute out faster^26^. Our investigation of the cholesterol effect on the thermotropic phase behavior of DPPC further supports the findings of Takechi-Haraya’s work (Fig. 3). Addition of cholesterol eliminated the pre-transition peak of DPPC and at 20 mol% cholesterol, the main phase transition broadened. The main phase transition was completely abolished at 40 mol% cholesterol. This shows that the amount of cholesterol can drastically change the thermotropic phase behavior of saturated lipid DPPC and disrupt the rigidity of the vesicle. Therefore, DPPC-Chol unloaded its drug depot faster than DPPC (Fig. 4) even though its encapsulation efficiency doubles DPPC’s.

The study on transport and clearance of (rigid) polycarbonate nanoparticles by Amrite *et al*.^10^ showed that the 20 nm particles were cleared fast from the episcleral surface, and were transported across the sclera to some extent. In comparison, 200 nm particles were found to be retained at the site of action for more than a month post injection (due to slower clearance of the particles compared to the smaller 20 nm particles). The particles used in our study are of sizes such that should be retained at the site of application and not subject to rapid clearance *in vivo*. In our study, we hypothesize that the liposomes are able to show some diffusion into the scleral tissue based on the malleability and fluidity of our particles compared to the rigid polycarbonate nanoparticles. The distribution of the liposomes may be different among the different types of liposomes, based on their phospholipid type, presence of cholesterol (bilayer rigidity) and charge. Compared to the positively charged liposomes, which remained at the episcleral region, the negatively charged liposomes were able to diffuse to some extent intrasclerally. One main limitation of our study is that it is an *ex vivo* transport study, and *in vivo*, factors such as clearance of drugs and carriers from the subconjunctival space will affect the transport and release duration of drugs post periocular administration. In addition, the duration of *ex vivo* experiments conducted were limited to a maximum of one week, due to scleral viability issues. Previous *ex vivo* experiments performed for long periods such as 6 days *in vitro* have indicated that under the experimental conditions, some swelling of collagen fibrils is expected compared to fresh sclera, as indicated by TEM micrographs but the scleral ultrastructure is preserved^33^. Moreover, any disintegration of the collagen fibril organization would have led to a sudden increase in permeability or a higher burst that would be visible in the daily release, which was not observed in our case. This observation was also consistent with results from other research groups who have conducted similar *in vitro* experiments for trans-scleral diffusion of solutes, in short and long-term studies^1^.

## Conclusions

In this study, ranibizumab encapsulated liposomes were studied in an *in vitro* and *ex vivo* setup. DPPC-DPPG attained a slower sustained release and a longer release duration of 21 days *in vitro* compared to the other formulations. The transport of negatively charged DPPC-DPPG across the sclera also showed favorable result with greater penetration. Although sustained transport of ranibizumab is achievable with neutral liposomes, they have a low degree of encapsulation efficiency. On the other hand, if the liposomes are unable to penetrate the sclera, they are likely to be washed away by the blood and lymphatic clearance mechanisms in an *in vivo* situation. In this case, the negatively charged liposome, DPPC-DPPG showed a higher degree of encapsulation and was able to penetrate into the sclera to achieve a depot effect. This technology has the potential to replace the current invasive intravitreal injections.

## Materials and Methods

### Materials

The lipids used: 1, 2-dipalmitoyl-sn-glycero-3-phosphocholine (DPPC), 1, 2-dipalmitoyl-3-trimethylammonium-propane (chloride salt) (DPTAP), 1, 2-dipalmitoyl-sn-glycero-3-phospho-(1’-rac-glycerol) (sodium salt) (DPPG), Rhodamine labeled phosphatidylethanolamine (Rho-PE) were purchased from Avanti Polar Lipids (Alabaster, AL, USA). Cholesterol was purchased from Sigma-Aldrich, USA. Polycarbonate filter membrane of size 0.2 μm, 0.08 μm and drain discs were purchased from Northern Lipids Inc., Canada. The water used in all the experiments was from a Mili-Q purification system with a resistivity of at least 18.2 ± 0.2 mΩ.cm. The solvents methanol and chloroform were of high-performance liquid chromatography (HPLC) grade (>99% purity) and were purchased from Tedia, USA. Phosphate Buffered Saline (PBS) was prepared from tablets purchased from Sigma-Aldrich, USA. Ranibizumab (Lucentis 10 mg/ml, Novartis AG, Switzerland) were kindly provided by our clinical collaborators and stored at 4 °C in the dark.

### Liposome Preparation and Use in the *Ex Vivo* Setup

Appropriate amounts of the lipid were weighed out into a round-bottom flask, and chloroform/methanol were added in the ratio of 2:1 (v/v) and manually shaken until the lipids have dissolved. The solvent was then completely evaporated using a rotary evaporator (IKA® RV 10, IKA® Werke GmbH & Co. KG, Staufen, Germany), connected to a water bath (IKA® MB 10 basic, IKA® Werke GmbH & Co. KG, Germany) maintained at 40 °C. The flask was rotated at 120 rpm under low pressure for an hour to eliminate any trace of residual solvent. The thin lipid film formed on the round bottom flask was hydrated with intrinsic buffer (10% trehalose, 0.01% tween and 13.5 mM histidine buffer pH 5.5), which leads to spontaneous formation of multilamellar vesicles (MLVs). The MLVs were then extruded sequentially through 0.2 µm and 0.08 µm polycarbonate filters fitted in a bench top extruder (Northern Lipids Inc., Canada), to obtain large unilamellar vesicles (LUVs). In the case of fluorescent lipid preparation, Lissamine-Rhodamine PE-labelled lipids were added (0.1 mol %) to the organic solvent mixture. For preparation of ranibizumab-loaded liposomes, the protein was introduced during the hydration of the dried thin film to form MLVs. After extrusion, the formulation was lyophilized. Prior to *in vitro* release or *ex vivo* transport experiments, the formulation was reconstituted using DI water. The hydrodynamic diameter and zeta potential of the formed liposomes were measured by dynamic light scattering (DLS) using a nanosizer instrument as described in Section 2.3.

### Liposome Characterization

The hydrodynamic diameter and polydispersity index as well as the zeta potential of the liposomal formulations, were determined using Malvern Zetasizer ZS™ (Malvern Instruments, UK). For size measurements, the formulations were diluted in DI water, the same samples were also used to analyze the zeta potential values.

### Determination of Encapsulation Efficiency

The encapsulation efficiency was determined by using ultracentrifugation method. Briefly, the liposomal solutions were centrifuged at 155,000 G for an hour using a benchtop Sorvall MTX 150 ultracentrifuge (Thermo Scientific, USA). The liposomal pellet obtained was broken using 1% Triton X-100 and ranibizumab was detected using ELISA (Enzyme-linked immunosorbent assay, SuperSignal® ELISA Pico Chemiluminescent Substrate 250 ml Kit) (see Section 2.6).

### *In Vitro* Release

For the *in vitro* release studies, 1 ml of drug loaded liposomal formulation was pipetted into a cellulose ester dialysis bag (100 kDa MWCO of membrane cut-off) and clipped at both ends using dialysis clips. The bags were placed in 20 ml of PBS buffer at pH 7.4, with 0.05% sodium azide, in amber colored glass bottles. The setup was placed in an orbital shaker (LM-400D, Yihder Co. Ltd, Taiwan), maintained at 37 °C at 50 rpm. At definite time intervals, 1 ml aliquots were taken from the release medium, stored in bovine serum albumin (BSA)-coated tubes and the dialysate was completely replaced with fresh PBS buffer. The release samples were analyzed using ELISA. Each experiment was repeated at least 3 times. At the end of the release studies, the dialysis bag was opened and the liposome formulation was broken with 1% Triton X-100 to obtain 100% loading.

### Enzyme-linked Immunosorbent Assay for Ranibizumab Quantification

The concentration of ranibizumab was analyzed by the ELISA method, slightly modified from previous reports^34^. High binding ELISA plates (Thermo Scientific, USA) were coated with 10 µg/ml of VEGF_165_ in 50 mM carbonate buffer pH 9.6, and incubated for 24 hours at 4 °C. The plates were washed three times with 0.05% Tween-20 in PBS, and the wells were blocked with 2% BSA (0.05% Tween-20 in PBS) overnight at 4 °C. After this, the plates were washed three times, and were ready for use. Appropriately diluted ranibizumab samples were aliquoted into the wells and incubated at RT for 2 hours with gentle shaking. The plate was washed five times with 0.05% Tween-20 in PBS pH 7.4 to remove unbound protein before loading the Peroxidase conjugated goat secondary antibody anti-human IgG (diluted 1:20,000 in PBS) which was incubated for 45 min at RT. The plates were again washed five times with 0.05% Tween-20 in PBS pH 7.4 and finally, ELISA chemiluminescent substrate was added. The reading was taken by measuring the luminescence of samples using a microplate reader (TECAN infinite M200, Switzerland). A standard calibration curve was included in every ELISA plate to account for any plate-to-plate variations. The detection limit of this assay was approximately 1.5 ng/ml. Results were represented as an average of triplicate measurements.

### *Ex Vivo* Studies

Porcine eyeballs were obtained from a local abattoir, with permission from the Agri-Food and Veterinary Association (AVA), Singapore. The adherent muscle tissue from the eye was gently removed, and the eye was excised at the limbus. The anterior chamber was cut out, and the vitreous gel was completely removed. The choroid and retinal layers were gently peeled away with forceps and the sclera was washed with PBS. The scleral cup was then cut into four equal parts and used either immediately or stored at −80 °C until use. The thickness of the scleral samples used for the *ex-vivo* experiments was measured with a digital caliper (Mitutoyo digital micrometer IP65 resolution 0.001 mm; Mitutoyo, Japan) and found to be 0.95 ± 0.25 mm.

**Figure 9:**
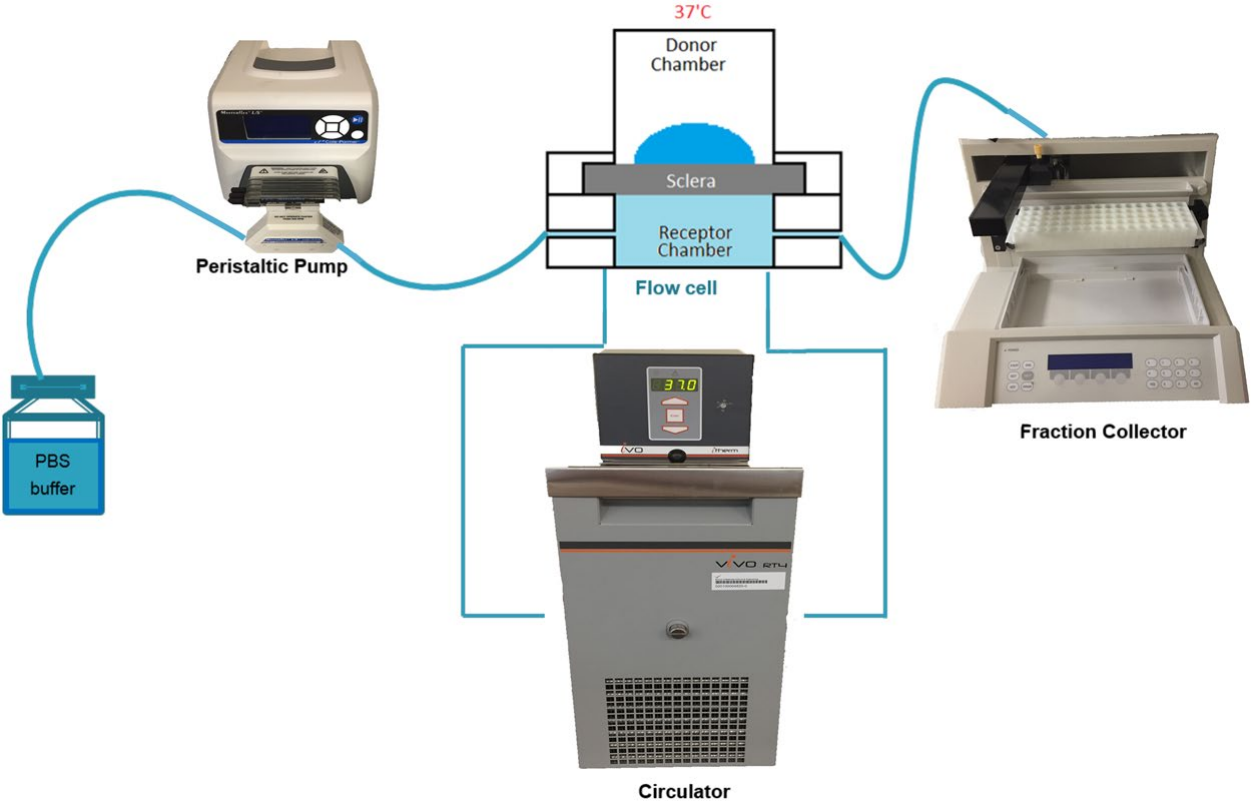
*Ex vivo* setup for transscleral transport. Plain ranibizumab and ranibizumab-loaded liposomes were loaded into donor chamber. The receiver chamber fractions were collected at regular time intervals.

We have designed an *ex vivo* setup for studying the transport of small molecules and carriers across the sclera, similar to those already reported in the literature^35^. Figure 9 depicts a schematic diagram of the setup used for our experiments. The frozen scleral tissue was thawed for about 30 minutes prior to each experiment. The thawed scleral tissue was cut into a circular section of 0.8 cm diameter and was mounted using a specially designed tissue-mounting ring, onto Ussing chambers (Navicyte, Harvard Apparatus). The episcleral side was facing the donor chamber and the vitreous side was facing the receiver chamber. This was done to mimic the subconjunctival drug delivery mode, where in trans-scleral drug delivery, drugs are released from the episcleral side and passed through the sclera to reach the vitreous. The chamber was maintained at 37 °C by a circulating water bath. The receiver chamber had a volume of 2.5 ml of PBS buffer. All bubbles between the underside of the tissue and the receiver chamber were carefully removed before the start of each experiment. The scleral was tested for leakage by introducing buffer solution in the donor chamber and checking for leakage for up to 30 minutes prior to each experiment. For transport experiments, 400 μl from donor solutions were collected in aliquots and kept in 4 °C. The scleral surface area was 0.5 cm^2^. The *ex vivo* setup, as well as the collection tubes and tubings used in the experiment, were coated with 1% BSA prior to the experiment, to avoid any antibody loss by non-specific adsorption to the walls.

Plain Ranibizumab and Ranibizumab loaded liposomes (DPPC, DPPC-Chol, DPPC-DPTAP and DPPC-DPPG) was loaded in the donor chamber. The receiver chamber fractions were collected every 24 hours, and replaced with equal volume of fresh buffer. Ranibizumab concentrations in the collected receiver chamber fractions were measured by ELISA, as described in Section 2.6.

### Tissue Processing and Cryosectioning

Following transport experiments with unloaded fluorescently labeled liposomes, localization and tissue distribution studies were carried out. The sclera was fixed in 4% Paraformaldehyde overnight. The next day, it was washed three times in PBS pH 7.4, embedded in Jung’s Tissue Freezing Media OCT compound and frozen at −80 °C. The frozen tissue blocks were then sectioned into smaller slices of 7 μm thickness using a Thermo HM550 cryostat microtome and were placed onto poly-lysine coated glass slides for imaging. The 7 μm thick tissue sections were imaged using a Carl Zeiss Axio Imager Z1 microscope (Zeiss, Germany). Bright-field images and fluorescence images (Ex 528 nm and Em 553 nm, red filter; Rh-PE) were acquired and overlaid to confirm and evaluate the extent of liposome penetration into the sclera. The Zeiss imaging software and Image J software were used to analyze the distance of penetration of the fluorescent liposomes into the sclera.

### Statistics

All experiments have been performed at least in triplicates, and the data presented are means ± standard deviation. Statistical data analysis was carried out using a Student’s t-test with P < 0.05 as the minimal level of significance.

## References

[1] Geroski, H. D. & Edelhauser, F. H. Drug Delivery for Posterior Segment Eye Disease. *Invest*. *Ophthalmol*. *Vis*. *Sci*. **41** (2000). 10752928

[2] Sampat KM, Garg SJ (2010). Complications of intravitreal injections. Curr. Opin. Ophthalmol..

[3] Raghava S, Hammond M, UB K (2004). Periocular routes for retinal drug delivery. Expert Opin. Drug Deliv..

[4] Kim, S. H. *et al*. Assessment of Subconjunctival and Intrascleral Drug Delivery to the Posterior Segment Using Dynamic Contrast-Enhanced Magnetic Resonance Imaging. *Invest*. *Ophthalmol*. *Vis*. *Sci*. **48** (2007). 10.1167/iovs.06-0670 17251481

[5] Ambati, J. *et al*. Diffusion of High Molecular Weight Compounds through Sclera. *Invest*. *Ophthalmol*. *Vis*. *Sci*. **41** (2000). 10752958

[6] Cheruvu, N. P. S. & Kompella, U. B. Bovine and Porcine Transscleral Solute Transport: Influence of Lipophilicity and the Choroid–Bruch’s Layer. *Invest*. *Ophthalmol*. *Vis*. *Sci*. **46** (2006). 10.1167/iovs.06-0404 PMC3324974 17003447

[7] Thakur A, Kadam RS, Kompella UB (2011). Influence of Drug Solubility and Lipophilicity on Transscleral Retinal Delivery of Six Corticosteroids. Drug Metabolism And Disposition.

[8] Kadam RS, Kompella UB (2010). Influence of Lipophilicity on Drug Partitioning into Sclera, Choroid-Retinal Pigment Epithelium, Retina, Trabecular Meshwork, and Optic Nerve. The Journal Of Pharmacology And Experimental Therapeutics.

[9] Wen H, Hao J, Li SK (2010). Influence of Permeant Lipophilicity on Permeation Across Human Sclera. Pharm. Res..

[10] Amrite AC, Kompella UB (2005). Size-dependent disposition of nanoparticles and microparticles following subconjunctival administration. J. Pharm. Pharmacol..

[11] Miao, H., Wu, B.-D., Tao, Y. & Li, X.-X. Diffusion of macromolecules through sclera. *Acta Ophthalmol*. (*Copenh*.) (2012). 10.1111/j.1755-3768.2012.02557.x 22998133

[12] Booth BA, Vidal Denham L, Bouhanik S, Jacob JT, JM H (2007). Sustained-release ophthalmic drug delivery systems for treatment of macular disorders: present and future applications. Drugs Aging.

[13] Lee SS, Robinson MR (2009). Novel Drug Delivery Systems for Retinal Diseases. Ophthalmic Res..

[14] Abrishami, M. *et al*. Preparation, Characterization, And *In Vivo* Evaluation Of Nanoliposomes-Encapsulated Bevacizumab (Avastin) For Intravitreal Administration. *Retina*, *J*. *Ret*. *Vit*. *Dis*. **29** (2009). 10.1097/IAE.0b013e3181a2f42a 19430280

[15] Bochot A, Fattal E (2012). Liposomes for intravitreal drug delivery: A state of the art. J. Control. Release.

[16] Cardillo JA, Souza-Filho AA, Oliveira AG (2006). Intravitreal Bioerudivel sustained-release triamcinolone microspheres system (RETAAC). Preliminary report of its potential usefulnes for the treatment of diabetic macular edema. Arch. Soc. Esp. Oftalmol..

[17] Elsaid N, Jackson TL, Elsaid Z, Alqathama A, Somavarapu S (2016). PLGA Microparticles Entrapping Chitosan-Based Nanoparticles for the Ocular Delivery of Ranibizumab. Mol. Pharm..

[18] K.Yandrapu S, Upadhyay AK, Petrash JM, Kompella UB (2013). Nanoparticles in Porous Microparticles Prepared by Supercritical Infusion and Pressure Quench Technology for Sustained Delivery of Bevacizumab. Mol. Pharm..

[19] Shelke NB, Kadam R, Tyagi P, Rao VR, Kompella UB (2011). Intravitreal Poly (L-lactide) Microparticles Sustain Retinal and Choroidal Delivery of TG-0054, a Hydrophilic Drug Intended for Neovascular Diseases. Drug Deliv. Transl. Res..

[20] Zhang L, Li Y, Zhang C, Wang Y, Song C (2009). Pharmacokinetics and tolerance study of intravitreal injection of dexamethasone-loaded nanoparticles in rabbits. Int. J. Nanomedicine.

[21] Kim SH, Lutz RJ, Wang NS, Robinson MR (2007). Transport Barriers in Transscleral Drug Delivery for Retinal Diseases. Ophthalmic Res..

[22] Natarajan JV (2014). Sustained drug release in nanomedicine: a long-acting nanocarrier-based formulation for glaucoma. ACS Nano.

[23] Natarajan, J. V. *et al*. Sustained Release of an Anti-Glaucoma Drug: Demonstration of Efficacy of a Liposomal Formulation in the Rabbit Eye. *PLoS One* **6** (2011). 10.1371/journal.pone.0024513 PMC3170360 21931735

[24] Barza, M., Baum, J. & Szoka, F. J. Pharmacokinetics of Subconjunctival Liposome-Encapsulated Gentamicin in Normal Rabbit Eyes. *Invest*. *Ophthalmol*. *Vis*. *Sci*. (1984). 6706512

[25] Li KS, Liddell MR, Wen H (2011). Effective electrophoretic mobilities and charges of anti-VEGF proteins determined by capillary zone electrophoresis. J. Pharm. Biomed. Anal..

[26] Takechi-Haraya, Y., Sakai-Kato, K. & Goda, Y. Membrane Rigidity Determined by Atomic Force Microscopy Is a Parameter of the Permeability of Liposomal Membranes to the Hydrophilic Compound Calcein. *AAPS PharmSciTech*, 1–7, 10.1208/s12249-016-0624-x (2016). 10.1208/s12249-016-0624-x 27645470

[27] McMullen TPW, Lewis RNAH, McElhaney RN (1993). Differential scanning calorimetric study of the effect of cholesterol on the thermotropic phase behavior of a homologous series of linear saturated phosphatidylcholines. Biochemistry.

[28] Barza M, Stuart M, Szoka F (1987). Effect of Size and Lipid Composition on the Pharmacokinetics of Intravitreal Liposomes. Invest. Ophthalmol. Vis. Sci..

[29] Fishman, P. H., Peymon, G. A. & Lesar, T. Intravitreal Liposome-Encapsulated Gentamicin in a Rabbit Model. *Invest*. *Ophthalmol*. *Vis*. *Sci*. **27** (1986). 3721788

[30] Honda, M. *et al*. Suppression of Choroidal Neovascularization by Intravitreal Injection of Liposomal SU5416. *Arch*. *Ophthalmol*. **129** (2011). 10.1001/archophthalmol.2011.12 21402988

[31] Kim, E. S. *et al*. Human Scleral Diffusion of Anticancer Drugs from Solution and Nanoparticle Formulation. *Pharm*. *Res*. **26** (2009). 10.1007/s11095-009-9835-0 19194787

[32] Briuglia M-L, Rotella C, McFarlane A, Lamprou DA (2015). Influence of cholesterol on liposome stability and on *in vitro* drug release. Drug Deliv. Transl. Res..

[33] Carrasquillo, K. G. *et al*. Controlled Delivery of the Anti-VEGF Aptamer EYE001 with Poly(lactic-co-glycolic)Acid Microspheres. *Invest*. *Ophthalmol*. *Vis*. *Sci*. **44** (2003). 10.1167/iovs.01-1156 12506087

[34] Moreno MR (2016). Study of stability and biophysical characterization of ranibizumab and aflibercept. Eur. J. Pharm. Biopharm..

[35] Cruysberg, L. P. J. *et al*. The Influence of Intraocular Pressure on the Transscleral Diffusion of High-Molecular-Weight Compounds. *Invest*. *Ophthalmol*. *Vis*. *Sci*. **46** (2005). 10.1167/iovs.04-1414 16186364

